# The impact of illness perception on self-transcendence in gastric cancer patients undergoing chemotherapy: the chain mediating effect of hope levels and coping styles

**DOI:** 10.3389/fpsyt.2025.1679697

**Published:** 2025-10-23

**Authors:** Qin Wang, Guoqin Ren, Li Sun, Xumiao Zhang, Hongxia Hua, Yanglin Gu, Su Qu

**Affiliations:** ^1^ Department Wuxi School of Medicine, Organization Jiangnan University, Wuxi, China; ^2^ Department Public Health, Jiangnan University Medical Center, Wuxi, China; ^3^ Department Oncology, Jiangnan University Medical Center, Wuxi, China; ^4^ Department Orthopedic, Jiangnan University Medical Center, Wuxi, China; ^5^ Department Administration office, Jiangnan University Medical Center, Wuxi, China

**Keywords:** chemotherapy patients, coping styles, gastric cancer, hope levels, illness perception, mediating effect, random forest model, self-transcendence

## Abstract

**Objective:**

To investigate the current status of illness perception, hope levels, coping styles, and self-transcendence among gastric cancer patients undergoing chemotherapy, and to explore the chain-mediating role of hope levels and coping styles in illness perception and self-transcendence.

**Methods:**

This study is a cross-sectional study. A convenience sampling method was used to select 507 gastric cancer patients undergoing chemotherapy from a tertiary hospital in Wuxi City, China, from October 2024 to May 2025. A questionnaire survey was conducted using Demographic Characteristics Questionnaire, Brief Illness Perception Questionnaire, Herth Hope Index, Medical Coping Modes Questionnaire, and Self-Transcendence Scale. Data were analyzed by SPSS 27.0, R Studio and Amos 24.0.

**Results:**

The mean scores for illness perception, hope levels, confrontation, avoidance, yield and self-transcendence were 42.79 ± 9.30, 34.10 ± 9.21, 24.50 ± 9.51, 20.69 ± 6.30, 14.70 ± 4.61 and 44.08 ± 10.38, respectively. The importance ranking of the random forest model was: illness perception, avoidance, confrontation, hope levels, self-rated health status and yield. The mediation model analysis indicated that illness perception had a significant direct effect on self-transcendence (confrontation, avoidance, and yield dimensions: 55.47%, 56.04%, 53.73%), the independent mediating effect of hope levels (30.90%, 32.13%, 32.05%), the independent mediating effect of coping styles (6.81%, 6.28%, 7.95%), and the chain mediating effect of hope levels and coping styles (6.81%, 5.85%, 6.27%).

**Conclusion:**

Illness perception directly influences self-transcendence in gastric cancer patients undergoing chemotherapy, indirectly influences self-transcendence through the mediating effects of hope levels, coping styles, and the chain mediating effect of hope levels and coping styles.

## 1 Introduction

Gastric cancer is a prevalent and life-threatening disease globally and represents one of the leading causes of cancer-related mortality in the word. According to the latest data released by the International Agency for Research on Cancer (IARC) (1), gastric cancer accounted for over 968,000 new cases and approximately 660,000 deaths globally in 2022, ranking as the fifth most common cancer and a leading cause of cancer-related mortality worldwide. The disease usually be discovered at an advanced stage, resulting in a significant disease burden and survival challenges (2). Moreover, gastric cancer patients undergoing chemotherapy frequently encounter considerable psychosocial challenges, including anxiety and depression. Research has indicated that gastrointestinal problems interact with psychological disorders such as depression through the microbiota-gut-brain axis mechanism (MGBA) (3). These issues not only impact patient adherence to therapy but are also intricately linked to quality of life (4). The NCCN emphasizes the importance of integrating mental health support into cancer management, listing psychological distress as the “sixth vital sign” (5). Consequently, examining the psychological adjustment mechanisms of gastric cancer patients, particularly identifying the factors that promote their positive psychological state and spiritual development, holds significant theoretical and practical significance. This study is founded on the stress-coping model (6), which highlights that when people encounter stressful situations, they first evaluate the stressors’ nature and importance cognitively, then produce emotional reactions and coping strategies, and ultimately affect their psychological and physical well-being. In gastric cancer patients undergoing chemotherapy, illness perception as the core of cognitive assessment, reflects patients’ comprehension of the illness, emotional experiences, and its consequences (7). Positive illness perception facilitates patients’ acceptance of their situation and fosters an optimistic outlook (7), whereas negative illness perception can engender frustration and helplessness, diminish hope, and prompt the adoption of maladaptive coping strategies (8). Hope, as a positive psychological resource, can enhance patients’ confidence and motivation in confronting their condition, facilitating their psychological recovery and adaptability (8). Coping strategies represent the specific behavioral strategies employed by patients and serve as a crucial link in the transition from cognition to mental health (9). Additionally, self-transcendence as a notion of psychological growth and spiritual elevation, underscores persons surpassing their limitations in the face of adversity to enhance their inner spiritual strength and sense of well-being (10). It performs as a crucial psychological resource for cancer patients to adapt to their illness and improve their quality of life (11). Previous studies indicates that self-transcendence levels exhibit a substantial negative correlation with patients’ illness perception (12) and a large positive correlation with hope levels (13). Nevertheless, these studies have predominantly concentrated on the impacts of singular psychological variables, failing to systematically integrate the underlying mechanisms among variables, particularly within the gastric cancer patient demographic, where there is an absence of chain-mediated research that encompasses cognitive, emotional, behavioral, and spiritual variables. Therefore, this study attempts to integrate illness perception (cognitive dimension), hope levels (emotional dimension), coping strategies (behavioral dimension), and self-transcendence (spiritual dimension) into an integrated theoretical framework, constructing a chain-mediated model to systematically investigate the mechanisms of psychological adaptation in gastric cancer patients undergoing chemotherapy. This study posits the following hypothesis based on the stress-coping model: Hypothesis 1: The level of illness perception in gastric cancer patients affects their degree of hope, consequently facilitating the attainment of self-transcendence. Positive cognition enhances patients’ confidence and optimistic expectations, thereby stimulating their level of hope and facilitating advanced psychological progress. Hypothesis 2: Illness perception indirectly influences self-transcendence by affecting patients’ coping strategies. Individuals with elevated cognitive levels are more inclined to adopt positive coping strategies, hence enhancing psychological adaption and self-transcendence; Hypothesis 3: Hope levels and coping strategies play a chain-mediated role in the process between illness perception and self-transcendence. Hope levels enhance patients’ coping resources and capabilities, fostering the implementation of positive coping strategies and ultimately facilitating self-transcendence. This study innovatively constructs a multi-level chain mediation model based on the stress-coping model to conduct an integrated analysis of illness perception, hope levels, coping strategies, and self-transcendence in gastric cancer patients undergoing chemotherapy. This not only enriches the theoretical framework of psychological adaptation in cancer patients but also provides scientific evidence for clinical nursing and psychological intervention, thereby enhancing the mental health and quality of life of cancer patients.

## 2 Methods

### 2.1 Study setting and sampling

Using a convenience sampling method, eligible gastric cancer patients undergoing chemotherapy were recruited from a tertiary hospital in Wuxi, China, between October 2024 and May 2025. Inclusion criteria: (1) Diagnosis of gastric cancer confirmed by histopathological and imaging examinations; (2) Expected survival time >6 months as clinically estimated by the department director according to gastric cancer treatment guidelines; (3) Age ≥18 years, with verbal/written communication abilities to complete questionnaires reliably; (4) Voluntary participation. Exclusion criteria: (1) Comorbid cognitive impairment, hearing/language barriers, or psychiatric disorders; (2) Physical frailty precluding questionnaire completion. Based on the rule of thumb for quantitative studies (14), the minimum required sample size was determined as 5~10 times the number of independent variables. With 31 predictor dimensions in the study scale, the target range was 155~310 participants. Accounting for a 10% invalid response rate, the minimum required sample was 341. To ensure robustness of structural equation modelling (SEM), sample sizes up to 600 enhance model stability (15), 524 questionnaires were collected. After exclusions, 507 participants were included in the final analysis. This study was approved by the Hospital Ethics Committee (Approval No. Y-305, 2024). All procedures adhered to the principles of voluntary participation, justice, confidentiality, and beneficence/non-maleficence.

### 2.2 Instruments

#### 2.2.1 Demographic characteristics questionnaire

This self-designed questionnaire comprised two sections: (1) Sociodemographic data, including gender, age, education, marital status, occupation, monthly, religious faith, living arrangements, group activity participation, personality traits, smoking, drinking, payment method and major event. (2) Clinical data: first consultation time, chronic, family history, treatment program, complications, self-rated health status, disease stage and metastasis status.

#### 2.2.2 Brief illness perception questionnaire

The questionnaire was developed by Broadbent et al. (16) This validated tool quantifies patients’ illness perceptions across cognitive, emotional and comprehension dimensions. The 9-item scale includes eight Likert-type items (0~10 points) and one open-ended question. Items 3, 4, and 7 are reverse-scored, yielding a total score of 0~80 (excluding the open response). Higher scores indicate stronger negative perceptions of disease threat. The Cronbach’s α in this study was 0.82, demonstrating good reliability and validity.

#### 2.2.3 Herth hope index

The questionnaire was developed by Herth (17). This 12-item scale assesses hope levels in cancer patients through three dimensions: positive attitudes toward reality and the future, proactive behaviors, and interpersonal connectedness. Items are rated on a 4-point Likert scale (1 = “strongly disagree” to 4 = “strongly agree”), with items 3 and 6 reverse-scored. Total scores classify hope levels as low (12~23), moderate (24~35), or high (36~48). The Cronbach’s α in this study was 0.85, demonstrating good reliability and validity.

#### 2.2.4 Medical coping modes questionnaire

The questionnaire was developed by Feifel et al. (18) consisted of 19 items. Chinese scholars Shen and Jiang (19) completed the systematic revision of the Chinese version, comprising 20 items with three dimensions: confrontation (8 items), yield (5 items) and avoidance (7 items). Eight items are reverse-scored using a 4-point Likert scale, widely applied in Chinese clinical settings. The overall score is 20 points. The higher the score in each area, the more inclined one is toward the related coping style. The Cronbach’s α in this study was 0.80, demonstrating good reliability and validity.

#### 2.2.5 Chinese version of the self-transcendence scale

The questionnaire was adapted from Reed (20)’s 15-item unidimensional scale by Zhang et al. (21) in 2014. This questionnaire measures self-transcendence using a 4-point Likert scale, with a total score ranging from 15 to 60. 1 to 4 points respectively represent “not at all,” “only a little,” “some,” and “a lot”. Scores ≤45 indicate low self-transcendence, while scores>45 reflect high levels. Higher scores denote greater self-transcendence capacity. The Cronbach’s α in this study was 0.88.

### 2.3 Data collection

Two trained nursing postgraduate students collected data using paper questionnaires with institutional consent. Researchers obtained written informed permission from eligible participants after explaining the study purpose, procedures, and confidentiality protocols. Participants independently completed the questionnaires. To assure response accuracy, researchers gave standardized verbal help in face-to-face interviews for persons with literacy or physical limitations. The entire process required approximately 10~30 minutes per participant. Completed questionnaires were immediately reviewed on-site to detect and rectify discrepancies or missing entries. Post-collection verification included rigorous checks for completeness, with exclusion criteria applied to questionnaires that contained more than 10% missing data. A total of 524 questionnaires were distributed, with 17 excluded due to incompleteness or ineligibility, resulting in a valid response rate of 96.76%.

### 2.4 Data analysis

Data underwent dual-entry verification by two researchers prior to analyze, data were analyzed using SPSS 27.0. Common method variance was assessed via Harman’s single-factor test. Descriptive statistics presented categorical variables as frequencies (percentages) and continuous variables as mean ± standard deviation (M ± SD). Pearson’s correlation analysis identified inter-variable relationships, after which statistically significant variables (*P* < 0.05) from univariate and correlation analyses were subjected to variable importance ranking through a random forest model in R Studio. Structural equation modeling (SEM) was constructed with AMOS 24.0 to test the chain mediating. Bootstrap was based on 5000 samples, and a 95% confidence interval (*CI*) that did not include 0 indicated a significant mediation effect. *P* < 0.05 was considered statistically significant. The absence of multicollinearity was confirmed by variance inflation factor (VIF) < 5 in regression models.

## 3 Results

### 3.1 Common method variance test

Harman’s single-factor test revealed 8 factors with eigenvalues >1, with the first factor explaining 37.05% of the variance (below the critical threshold of 40%) (22), indicating no substantial common method bias.

### 3.2 Self-transcendence scores across patient characteristics

Among the 507 gastric cancer patients undergoing chemotherapy, univariate analysis of self-transcendence scores identified statistically significant differences (*P* < 0.05) by age, education, marital status, occupation, monthly, religious faith, living arrangements, group activity participation, personality traits, payment method, complications, and self-rated health status (All detailed in **Table 1**).

**Table 1:** Social-demographic characteristics of participants and comparison of different variables on Self-Transcendence (N = 507).

Variable	Category	Frequency (n)	Percentage (%)	Mean ± SD	*F*/*t*	*P*
Gender	Male	297	58.6	1.410 ± 0.493	0.902	.368
	Female	210	41.1			
Age(years)	18-60	99	19.5	2.350 ± 0.870	5.750	.001^a^
	60-70	167	32.9			
	70-80	206	40.6			
	≥80	35	6.9			
Education	Primary school or below	108	21.3	2.220 ± 0.868	2.846	.037^b^
	Junior high school	218	43.0			
	Senior high school	142	28.0			
	College or above	39	7.7			
Marital Status	Single	2	0.4	2.470 ± 0.796	5.096	.002^a^
	Married	361	71.2			
	Divorced	49	9.7			
	Widowed	95	18.7			
Occupation	Farmer	127	25.0	2.260 ± 1.000	5.442	.001^a^
	Worker	195	38.5			
	Public institution	112	22.1			
	Corporate employee	71	14.0			
	Other	2	0.4			
Monthly Income (CNY)	≤2000	116	22.9	2.360 ± 0.997	7.491	.001^a^
	2001~4000	168	33.1			
	4001~6000	146	28.8			
	>6000	77	15.2			
Religious Faith	Yes	83	16.4	1.840 ± 0.370	3.881	.001^a^
	No	424	83.6			
Living Arrangements	Living alone	53	10.5	2.960 ± 1.042	6.493	.001^a^
	spouse	116	22.9			
	children	149	29.4			
	spouse and children	178	35.1			
	relatives/friends	11	2.2			
Group Activity Participation	Yes	270	53.3	1.470 ± 0.499	2.895	.004^a^
	No	237	46.7			
Personality Traits	Extroverted	287	56.6	1.430 ± 0.496	5.778	.001^a^
	Introverted	220	43.4			
Smoking	Yes	110	21.7	2.040 ± 0.688	0.099	0.906
	No	267	52.7			
	Have given up	130	25.6			
Drinking	Yes	102	20.1	2.040 ± 0.664	2.088	0.125
	No	283	55.8			
	Have given up	122	24.1			
Payment Method	Employee insurance	305	60.2	1.510 ± 0.740	12.228	.001^a^
	Resident insurance	167	32.9			
	Out-of-pocket	15	3.0			
	Other	20	3.9			
Major Event	Yes	243	47.9	1.520 ± 0.500	0.074	0.941
	No	264	52.1			
First Consultation time(years)	≤1	292	57.6	1.490 ± 0.617	4.348	0.053
	1 ~5	182	35.9			
	≥5	33	6.5			
Chronic	0	127	25.0	2.190 ± 0.898	0.973	0.405
	1	195	38.5			
	2	147	29.0			
	≥3	49	9.7			
Family History	Yes	90	17.8	1.820 ± 0.382	1.420	0.156
	No	419	82.2			
complications	0~1	203	40.0	1.790 ± 0.739	26.936	.001^a^
	2~3	208	41.0			
	>3	97	18.9			
Treatment Program	operation	127	25.0	2.140 ± 0.789	1.558	0.212
	chemotherapy	182	35.9			
	Operation + chemotherapy	198	39.1			
Self-Rated Health Status	Good	75	14.8	3.140 ± 0.684	35.415	.001^a^
	Fair	299	59.0			
	Poor	120	23.7			
	Very poor	13	2.6			
Disease Stage	≤II	139	27.4	1.900 ± 0.662	25.232	.001^a^
	III	280	55.2			
	≥IV	88	17.4			
Metastasis Status	Yes	315	62.1	1.380 ± 0.486	5.913	.001^a^
	No	192	37.9			

^a^: P < 0.01, ^b^: P < 0.05.

### 3.3 Descriptive statistics and correlations

According to the study results, the mean score for illness perception, hope levels, confrontation, avoidance, yield and self-transcendence were 42.79 ± 9.30, 34.10 ± 9.21, 24.50 ± 9.51, 20.69 ± 6.30, 14.70 ± 4.61 and 44.08 ± 10.38, respectively. Pearson’s correlation analysis showed that self-transcendence was positively associated with hope levels (r = 0.538, *P* < 0.01) and confrontation (r = 0.516, *P* < 0.01) but negatively associated with illness perception (r = -0.537, *P* < 0.01), avoidance (r = -0.503, *P* < 0.01), and yield (r = -0.489, *P* < 0.01); hope levels was positively associated with confrontation (r = 0.481, *P* < 0.01) yet negatively associated with illness perception (r = -0.579, *P* < 0.01), avoidance (r = -0.391, *P* < 0.01), and yield (r = -0.388, *P* < 0.01); illness perception was negatively associated with confrontation (r = -0.457, P < 0.01) but positively associated with avoidance (r = 0.479, *P* < 0.01) and yield (r = 0.498, *P* < 0.01). (All detailed in **Table 2**).

**Table 2:** Correlation analysis between self-transcendence and influencing factors.

Variable	1	2	3	4	5	6	7	8	9	10	11	12
1	1											
2	.540**	1										
3	.493**	.415**	1									
4	.947**	.753**	.640**	1								
5	-.411**	-.385**	-.374**	-.468**	1							
6	-.358**	-.385**	-.349**	-.427**	.610**	1						
7	-.421**	-.380**	-.405**	-.479**	.582**	.595**	1					
8	-.464**	-.448**	-.439**	-.535**	.859**	.861**	.845**	1					
9	.401**	.423**	.376**	.473**	-.462**	-.426**	-.487**	-.536**	1				
10	-.384**	-.387**	-.378**	-.450**	.488**	.454**	.391**	.521**	-.304**	1			
11	.443**	.421**	.362**	.500**	-.448**	-.452**	-.493**	-.543**	.714**	-.333**	1	
12	-.462**	-.474**	-.454**	-.545**	.487**	.504**	.494**	.579**	-.517**	.517**	-.530**	1

**. At the 0.01 level (two-tailed), the correlation is significant.

1 Cognitive dimensions, 2 Emotional dimensions, 3 Comprehension dimensions, 4 Illness Perception, 5 Positive attitudes towards reality and the future, 6 Taking positive action, 7 Maintaining close contact with others, 8 Hope levels, 9 Avoidance, 10 Confrontation, 11 Yield, 12 Self-transcendence.

### 3.4 Variable importance ranking of determinants influencing self-transcendence in gastric cancer patients undergoing chemotherapy

**Figure 1:**
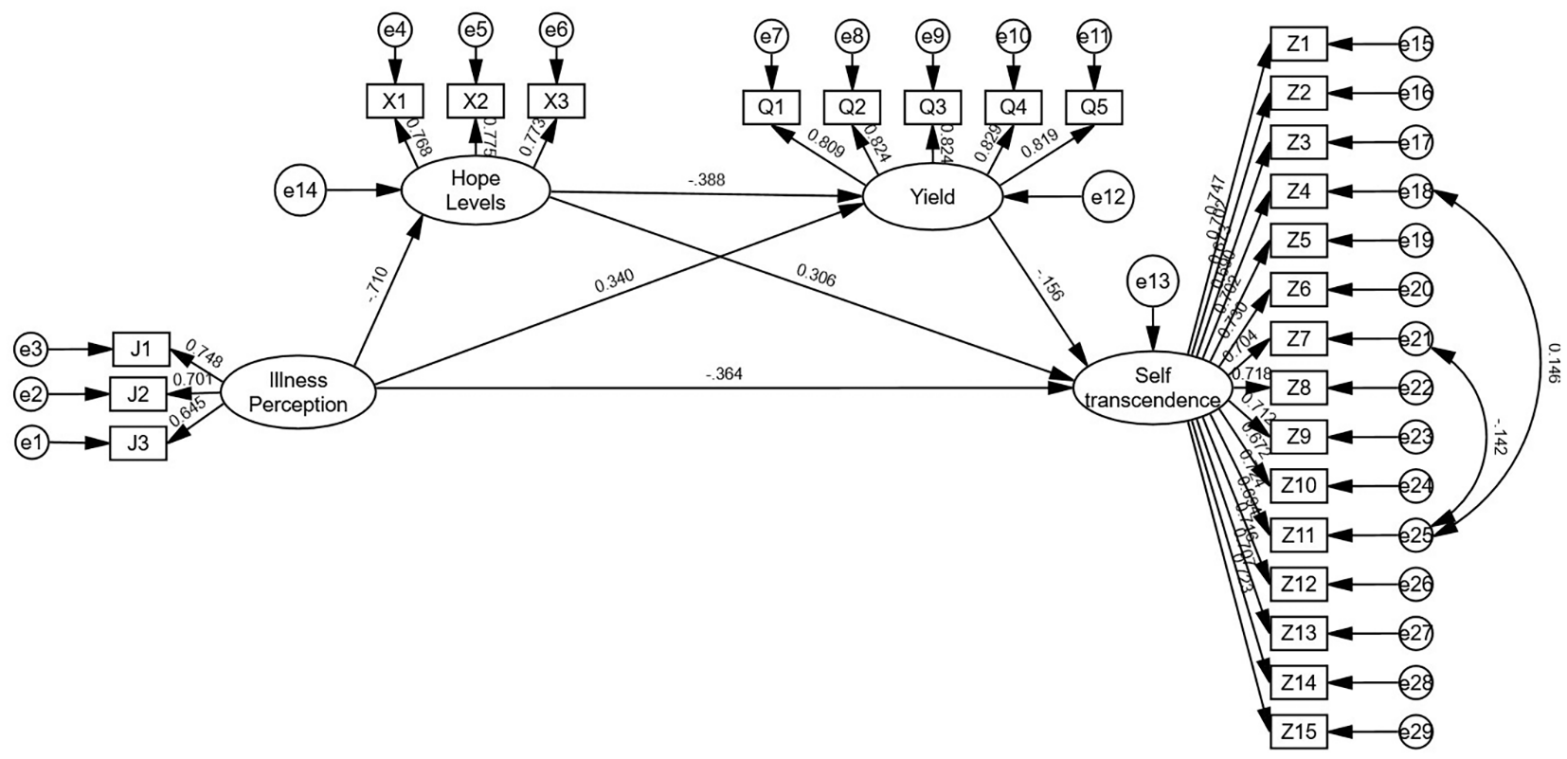
The chain mediating model 3(yield) of research.

Using self-transcendence as the dependent variable, a random forest model was implemented in R Studio incorporating 19 variables identified as statistically significant (*P* < 0.05) from prior univariate and correlation analyses. Variable importance was quantified by %Inc MSE (Percentage Increase in Mean Squared Error), where higher values indicate greater predictive importance (23). Key determinants ranked in descending order of importance were: illness perception, avoidance dimensions, confrontation dimensions, hope levels, self-rated health status, and yield dimensions (All detailed in **Figure 1**).

### 3.5 The chained mediating role of hope levels and coping styles in the relationship between illness perception and self-transcendence in gastric cancer patients undergoing chemotherapy

**Figure 2:**
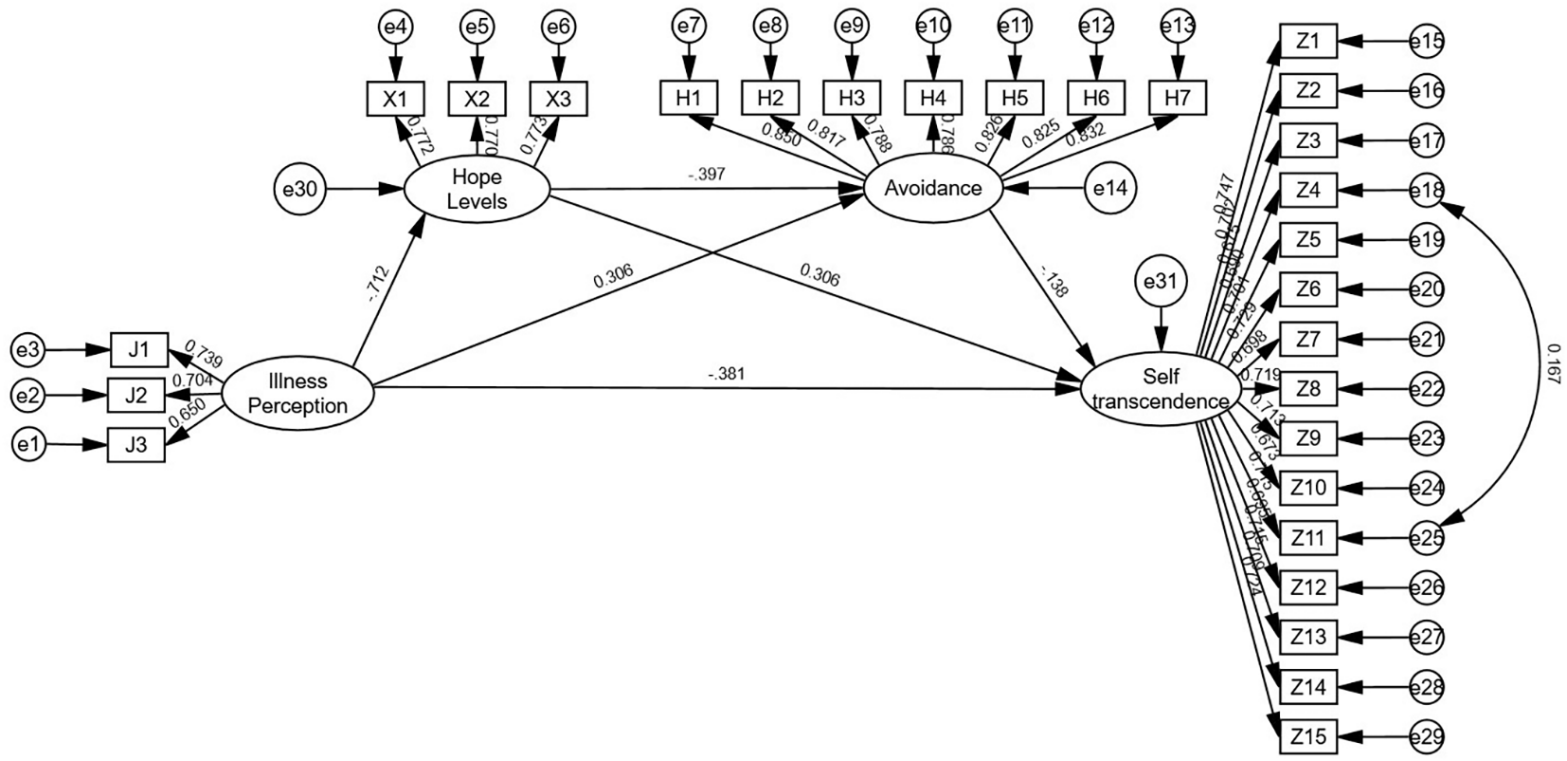
The chain mediating model 2(avoidance) of research.

**Figure 3:**
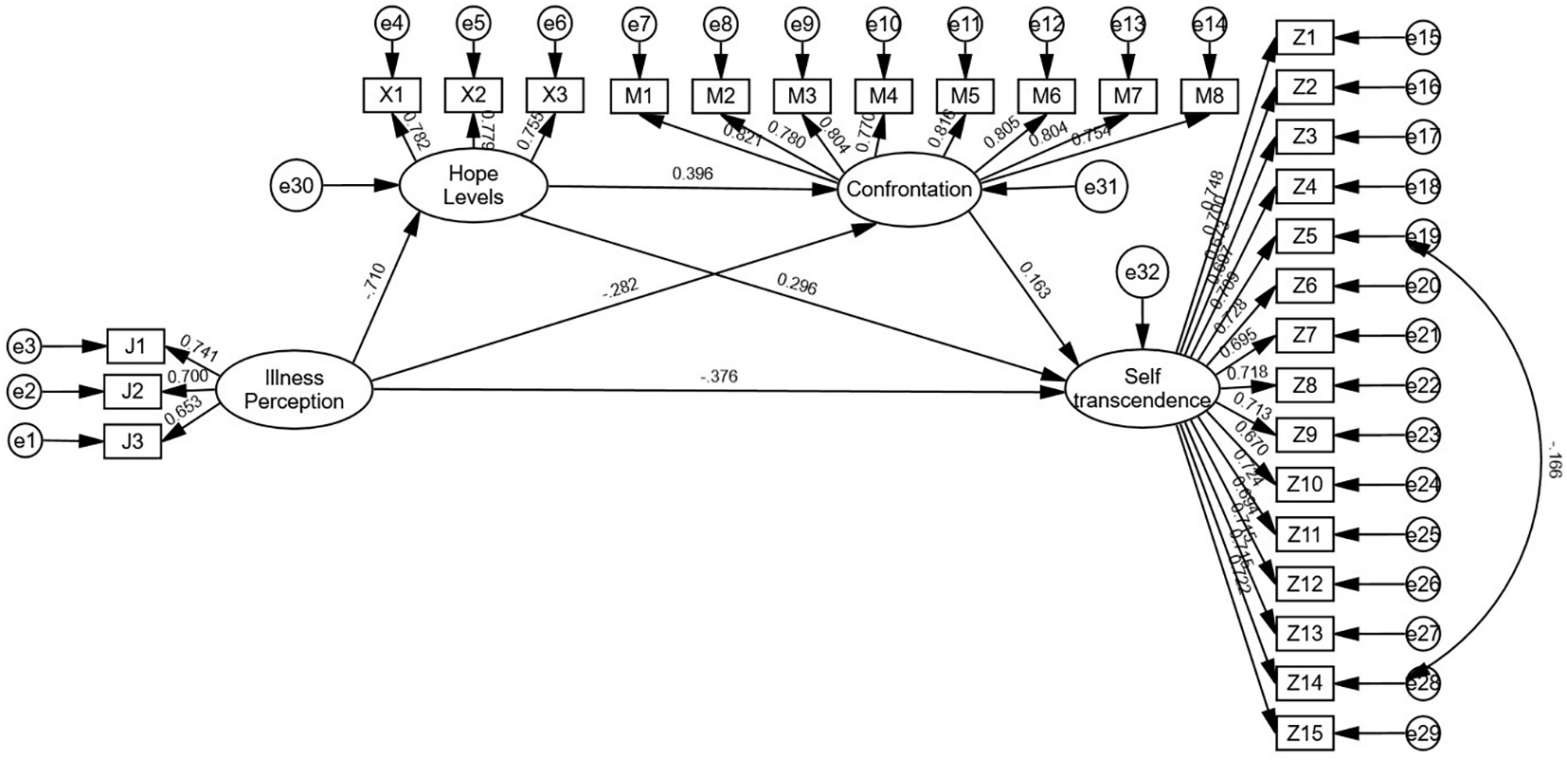
The chain mediating model 1(confrontation) of research.

**Figure 4:**
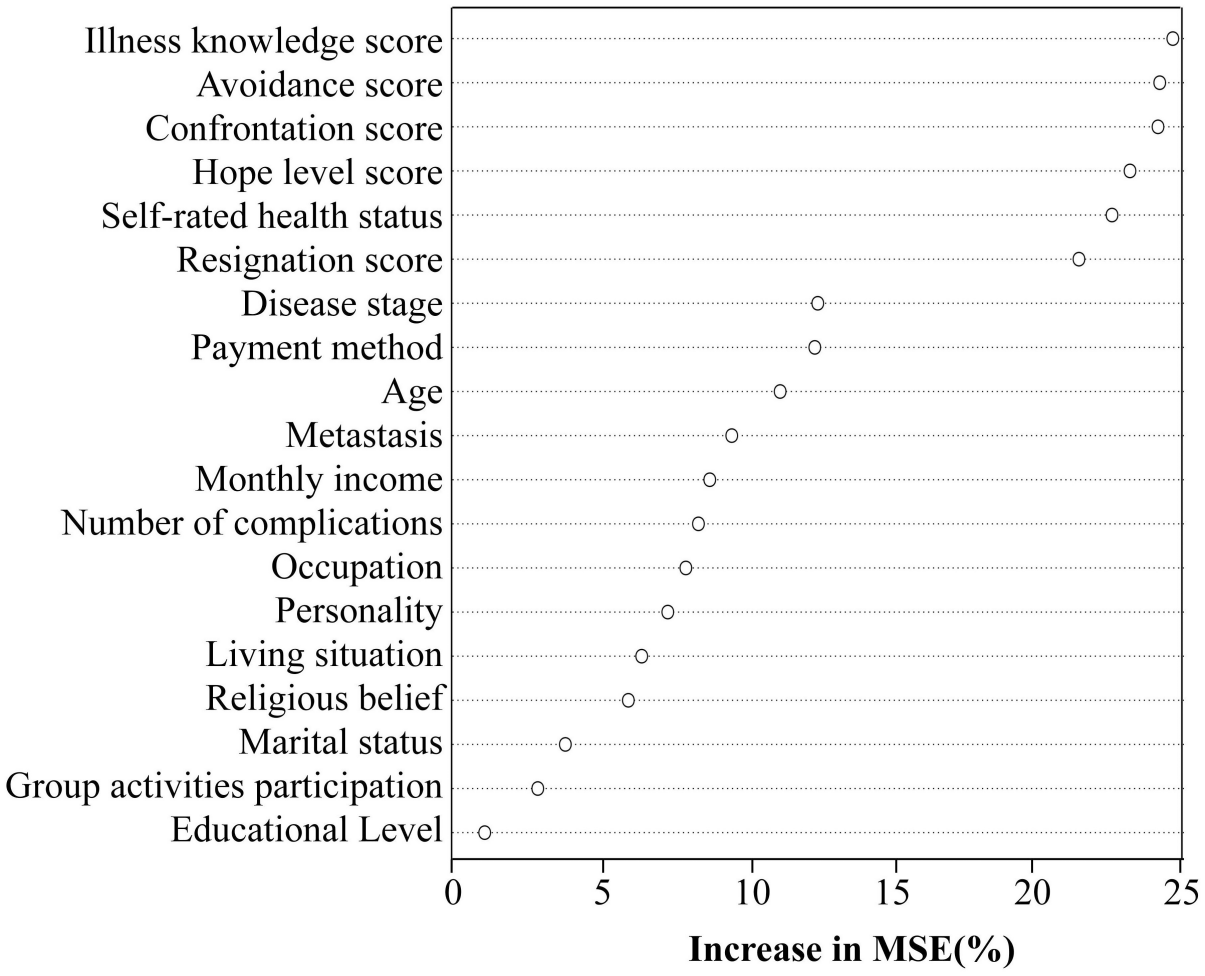
Variable importance ranking of influencing factors for self-transcendence in gastric cancer patients undergoing chemotherapy.

The hypotheses of structural equation modeling were constructed with illness perception as the independent variable, self-transcendence as the dependent variable, hope levels and coping styles (confrontation, avoidance, yield) as the mediator variables. Regression equations 1, 2, and 3 were constructed. All models demonstrated good fit. See **Figures 2**–**4**. for details.

Model 1 (confrontation): χ²/df = 1.329, RMSEA = 0.026, GFI = 0.939, AGFI = 0.928, CFI = 0.985, IFI = 0.985. Illness perception negatively predicted hope levels (β = -0.710, *P* < 0.01), confrontation (β = -0.282, *P* < 0.01), and self-transcendence (β = -0.376, *P* < 0.01); hope levels positively predicted confrontation (β = 0.396, *P* < 0.01) and self-transcendence (β = 0.296, *P* < 0.01); confrontation positively predicted self-transcendence (β = 0.163, *P* < 0.01).

Model 2 (avoidance): χ²/df = 1.128, RMSEA = 0.016, GFI = 0.948, AGFI = 0.939, CFI = 0.995, IFI = 0.995. Illness perception positively predicted avoidance (β = 0.360, *P* < 0.01) while negatively predicting hope levels (β = -0.712, *P* < 0.01) and self-transcendence (β = -0.381, *P* < 0.01); hope levels negatively predicted avoidance (β = -0.397, *P* < 0.01) and positively predicted self-transcendence (β = 0.306, *P* < 0.01); avoidance negatively predicted self-transcendence (β = -0.138, *P* < 0.01).

Model 3 (yield): χ²/df = 1.085, RMSEA = 0.013, GFI = 0.955, AGFI = 0.945, CFI = 0.996, IFI = 0.996. Illness perception positively predicted yield (β = 0.340, *P* < 0.01) and negatively predicted hope levels (β = -0.710, *P* < 0.01) and self-transcendence (β = -0.364, *P* < 0.01); hope levels negatively predicted yield (β = -0.388, *P* < 0.01) and positively predicted self-transcendence (β = 0.306, *P* < 0.01); yield negatively predicted self-transcendence (β = -0.156, *P* < 0.01).

As detailed in **Table 3**, Bootstrap analyses revealed significant partial mediation effects (all 95% confidence intervals excluded 0). For Model 1, the direct effect accounted for 55.47% of the total effect, while total indirect effects represented 44.53%, with the chained mediation of hope levels and confrontation dimensions contributing 6.81%. In Model 2, the direct effect accounted for 56.04% of the total effect, total indirect effects 43.96%, and hope levels and avoidance dimensions chained mediation contributed to 5.85%. Model 3 showed a direct effect proportion of 53.73%, total indirect effects 46.27%, and hope levels and yield dimensions chained mediation 6.27%. These findings confirm that hope levels combined with confrontation, avoidance, and yield dimensions partially mediate the relationship between illness perception and self-transcendence. The structural pathways are illustrated in **Tables 3**.

**Table 3:** Analysis of the mediating effect of hope levels and coping styles in gastric cancer chemotherapy patients between illness perception and self-transcendence.

Model	Path	Effect size	Boot SE	95%CI		Relative mediation effect%
				LLCI	ULCI	
Confrontation	Illness perception→Hope levels→Self-transcendence	-0.127	0.054	-0.243	-0.034	30.90
	Illness perception→Confrontation→Self-transcendence	-0.028	0.013	-0.064	-0.008	6.81
	Illness perception→Hope levels→Confrontation→Self-transcendence	-0.028	0.015	-0.069	-0.005	6.81
	Indirect effect	0.183	0.057	-0.293	-0.076	44.53
	Direct effect	0.228	0.076	-0.399	-0.107	55.47
	Total effect	-0.411	0.042	-0.495	-0.327	
Avoidance	Illness perception→Hope levels→Self-transcendence	-0.133	0.058	-0.250	-0.023	32.13
	Illness perception→Avoidance→Self-transcendence	-0.026	0.012	-0.055	-0.005	6.28
	Illness perception→Hope levels→Avoidance→Self-transcendence	-0.024	0.012	-0.054	-0.005	5.85
	Indirect effect	-0.182	0.061	-0.308	-.0.072	43.96
	Direct effect	-0.232	0.081	-0.407	-0.095	56.04
	Total effect	-0.414	0.043	-0.500	-0.330	
Yield	Illness perception→Hope levels→Self-transcendence	-0.133	0.058	-0.253	-0.027	32.05
	Illness perception→Yield→Self-transcendence	-0.033	0.017	-0.076	-0.005	7.95
	Illness perception→Hope levels→Yield→Self-transcendence	-0.026	0.014	-0.062	-0.005	6.27
	Indirect effect	-0.192	0.060	-0.315	-0.082	46.27
	Direct effect	-0.223	0.081	-0.404	-0.088	53.73
	Total effect	-0.415	0.044	-0.504	-0.330	

## 4 Discussion

### 4.1 Current analysis of illness perception, hope levels, coping styles, and self-transcendence in gastric cancer chemotherapy patients

The illness perception of gastric cancer patients undergoing chemotherapy was 42.79 ± 9.30, indicating a moderate degree, in accordance with Liu et al. (24)’s research. The intricate pathophysiology, multifaceted treatment regimens, and considerable side effects of this malignancy impede thorough illness comprehension, fostering anxiety, fear, and cognitive distortion. Research indicates that 76.2% of cognitively challenged patients suffer from simultaneous weariness and physical deterioration, exacerbating negative cognitive through symptom synergy (25). The objective reality of a mere 20% five-year survival rate engenders therapeutic skepticism (26), cultivating a negative perception that “treatment may be ineffective”. Patients predominantly depend on physician consultations for information, but time limitations, inadequate communication, and health literacy obstacles often engender helplessness, diminishing proactive disease education engagement. Insufficient social support networks (family, friends, rehabilitation institutions) compound emotional and informational deficits, reinforcing negative cognitions (27). In traditional cultural contexts, patients often refrain from discussing cancer owing to fear, and psychological defense mechanisms lead them to eschew information about the disease, hindering cognitive improvement and resulting in elevated levels of negative cognition.

The hope levels of gastric cancer patients undergoing chemotherapy was 34.10 ± 9.21, indicating a moderate level, in accordance with Li et al. (28)’s research. The level of hope fundamentally reflects the dynamic equilibrium between the threat of disease and the capacity for survival resilience. While chemotherapy postpones disease progression and alleviates symptoms, its non-curative nature diminishes patients’ expectations for complete recovery, maintaining their hope at a moderate level (29). Furthermore, high-frequency toxicities (nausea, myelosuppression, neuropathy) and prevalent malnutrition collectively suppress hope elevation. 75% of gastric cancer patients undergoing chemotherapy experience cancer pain, which severely affects their quality of life and serving as an independent risk factor for psychological distress and low hope levels (30). Inadequate family resilience is detrimental to enhancing patients’ confidence in disease treatment (31).

The confrontation dimension score for gastric cancer patients undergoing chemotherapy for was 24.50 ± 6.51, the avoidance dimension score was 20.69 ± 6.30, and the yield dimension score was 14.70 ± 4.61. The scores for each dimension exceeded those reported in Ma et al. (32)’s research, potentially attributable to differences in coping styles arising from variations in disease type. Research indicates that patients with high self-efficacy and psychological resilience prefer confrontation. Support from family and friends, along with access to high-quality medical care, significantly contributes to patients’ effective coping with illness. However, patients with more severe adverse reactions exhibit higher levels of avoidance and yield behaviors. The perception of illness and coping styles are significantly influenced by cultural background. Cross-cultural analyses indicate that Eastern patients exhibit greater emotional restraint than Western counterparts, manifesting as higher avoidance/yield and lower confrontation scores.

The self-transcendence score of gastric cancer patients undergoing chemotherapy was 44.08 ± 10.38, indicating a low level of self-transcendence. However, compared with Bozkurt and Yildirim (33)’s research, the self-transcendence levels of gastric cancer patients undergoing chemotherapy have improved, potentially attributable to extensive adoption of national medical insurance policies, leading to a significant percentage of participants in this study qualifying for medical insurance reimbursement (93.1%). The importance ranking of the random forest model indicates that self-rated health status is the fifth most significant factor influencing self-transcendence. Patients who perceive their health status as good often exhibit a more positive mindset, which is associated with positive intrinsic motivation, thereby promoting self-transcendence at the spiritual level. The most distinctive characteristic of gastric cancer patients, compared to other cancer types is the prominence of digestive symptoms, such as abdominal pain, fasting and strong appetite but poor digestion, which directly undermine patients’ appetite Based on the traditional Chinese belief that “inability to eat portends shortened survival”, patients experience heightened psychological distress, subjectively perceive their health status as poor, and concentrate more on physical discomfort and limitations. Consequently, their motivation and capacity for self-transcendence diminish, resulting in lower levels of self-transcendence.

### 4.2 Illness perception directly influences self-transcendence in gastric cancer chemotherapy patients

This study indicates that illness perception directly affects self-transcendence in gastric cancer patients undergoing chemotherapy, with effect proportions for the confrontation, avoidance, and yield dimensions were 55.47%, 56.04%, and 53.73%, respectively. According to Reed’s Theory of Self-Transcendence (34), illness perception is patients’ subjective interpretation and evaluation of disease-related information, wherein positive illness perception appraisals facilitate psychological and spiritual transcendence through their experience of disease. Patients with gastric cancer receiving chemotherapy experience significant physiological and social challenges, including nutritional deficiencies, digestive issues, and social isolation, necessitating a reconstruction of their understanding of illness and life-meaning. Upon acknowledging the fragility of life, patients may paradoxically intensify their pursuit of self- transcendence concepts, including “spiritual connection” and “meaning of life,” resulting in a significant bio-psychosocial coupling effect. This corresponds with the theoretical principle of “sudden transcendence that may occur when a predicament reaches a critical point” in self-transcendence theory (35). The persistent family-oriented culture in China, which regards the family as central to both individual and social existence, further catalyzes this transformation. Family responsibilities, including the commitment to “live for one’s family” and “actively seeking treatment to alleviate family concerns”, also act as a catalyst for the transformation of illness perception into self-transcendence (36), thereby enhancing one’s inner strength to face challenges. Therefore, illness perception has a strong effect on the direct impact on the level of self-transcendence. Healthcare professionals can enhance the illness perception levels of gastric cancer patients through implementing family-centered psychological support intervention programs (37) or cognitive-behavioral therapy (38). This involves educating patients on disease-related knowledge, introducing preventive measures and management strategies for chemotherapy-related side effects, distributing informational brochures on symptom management, enhancing coping abilities, alleviating fears, minimizing the disease’s impact on daily life, and ultimately improving overall quality of life (39).

### 4.3 The mediating role of hope level between illness perception and self-transcendence in gastric cancer chemotherapy patients

Mediation analysis indicated that hope levels were negatively correlated with illness perception levels, which partially mediated the relationship between illness perception and self-transcendence. The mediation effect values for the confrontation, avoidance, and yield dimensions were 30.90%, 32.13%, and 32.05%, respectively, with the highest effect sizes observed in the mediation paths across all dimensions. Recent research tends to utilize hope levels as a bridge to explore the relationship between illness perception and advanced psychological adaptation in cancer patients (31, 40). Hope as a psychological variable with positive orientation and intrinsic motivational effects, can effectively transform illness perception into spiritual self-transcendence (41), thus exhibiting significant and high effect values in the chain mediating process. Clear illness perception enables patients to accurately evaluate their disease status, mitigate uncertainty and fear, thereby enhancing hope and psychological resilience (42). Zhang et al. (43)’s research indicates that future studies should prioritize enhancing hope and reducing patients’ negative illness perceptions when developing targeted intervention strategies. Healthcare professionals should assist patients in establishing scientific and reasonable illness perception, perform regular mental health evaluations, promptly identify patients’ anxiety, depression, and despair, and enhance patients’ sense of hope through cognitive-behavioral therapy, positive psychological interventions, and other strategies (44). Stage-specific goals should be established for patients, and both patients and their families should be encouraged to participate in support groups or psychological counseling activities, while fostering a supportive environment. By following the chain of “goal-driven—meaning-giving—action-reinforcing,” the hope levels of gastric cancer patients undergoing chemotherapy can be improved (45), thereby enhancing disease cognition and self-transcendence levels.

### 4.4 The mediating role of coping styles between illness perception and self-transcendence in gastric cancer chemotherapy patients

Mediation analysis indicated that coping styles partially mediated the relationship between illness perception and self-transcendence, with effect sizes of 6.81%, 6.28%, and 7.95% for the dimensions of confrontation, avoidance, and yield, respectively. Patients’ illness perceptions directly influence their selection of coping styles, ultimately affects treatment outcomes (46). Correct and positive understanding of the illness enables patients to adopt positive coping strategies, such as seeking social support and arranging treatment appropriately, which in turn alleviates anxiety and depression. Incorrect or negative comprehension of the illness frequently results in patients adopting negative coping strategies such as avoidance or yield, which affects treatment outcomes and mental health. Embracing a positive attitude aids patients in managing the psychological stress linked to their illness effectively and preventing chemotherapy-related complications, enhance a sense of internal control and meaning, as well as to promote self-transcendence. Avoidance and yield dimensions may exacerbate patients’ fear of the unknown elements of chemotherapy, undermine their confidence in managing the disease, and lead to decreased adherence to treatment. Research by Segerstrom and Miller (47) indicates that negative coping mechanisms regulate neuroendocrine mechanisms, leading to sustained activation of the HPA axis, accelerated decline in immune function, and a vicious cycle of physiological and psychological issues, which is detrimental to achieving self-transcendence. Healthcare providers ought to deliver personalized education to gastric cancer patients undergoing chemotherapy. For those who maintain a positive attitude, actively provide visual explanations of the tumor shrinkage process and encourage patients to share their cancer-fighting experiences (48). For those exhibiting a negative attitude, self-disclosure interventions should be implemented to encourage them to share their treatment experiences and alleviate psychological distress (49), and through re-evaluating of their condition, rebuild their sense of control over it, thereby facilitating behavioral change and fostering self-transcendence.

### 4.5 Chained mediating effects of hope level and coping styles between illness perception and self-transcendence

The chain-mediated effect values of hope levels and the dimensions of confrontation, avoidance, and yield between patients’ illness perception and self-transcendence were 6.81%, 5.85%, and 6.27%, respectively. Hope levels exhibited a positive correlation with the confrontation dimension and a negative correlation with the avoidance, and yield dimensions, consistent with the findings of Yildizel et al. (50). The Transactional Model of Stress and Coping emphasizes that individuals mobilize their own resources to implement suitable coping styles after facing stressful events. Hope level, as an individual’s coping resource, directly influences patients’ reaction to stressful events. Patients exhibiting elevated levels of hope are more inclined to pursue solutions, stimulate intrinsic motivation, and thus tend to adopt positive coping strategies (43). This suggests that a patient’s positive cognition regarding gastric cancer chemotherapy correlates with higher levels of hope, a more favorable attitude toward the disease, and an increased likelihood of achieving self-transcendence. Therefore, healthcare providers should actively correct patients’ cognitive biases prior to chemotherapy, implement mindfulness interventions to enhance patients’ hope levels, thereby promoting their ability to appropriately confront chemotherapy effectively (51), ultimately achieving self-transcendence, and improving the quality of life for gastric cancer patients undergoing chemotherapy.

## 5 Conclusions

The illness perception of gastric cancer patients undergoing chemotherapy can directly influence self-transcendence and indirectly influence it through hope levels, coping styles, and their chain mediating effects. The current level of self-transcendence among gastric cancer patients undergoing chemotherapy is relatively low. Therefore, healthcare professionals should identify patients with low self-transcendence levels early on and enhance their understanding and acceptance of disease through cognitive and hope-related interventions. This will help gastric cancer patients undergoing chemotherapy achieve new breakthroughs in both psychological and physiological aspects, ultimately enhancing their overall quality of life.

### 5.1 Limitations

This study only investigated the relationships among the four factors through a cross-sectional study and included patients from a single hospital, resulting in restricted sample representativeness and extrapolation. In addition, the measurement results of scales may be subject to subjective bias and may be influenced by the emotional state of the subjects. Future studies should be conducted in a multi-center, large-scale setting, incorporating objective outcomes as endpoints to reduce bias, and explore the possibility of other potential pathways besides the results of this study. Our study was conducted in China within a specific cultural setting, and the expression and measurement of these psychological constructs might differ across cultures. Future cross-cultural comparative studies are warranted to enhance our understanding of these psychological mechanisms among gastric cancer patients.

## Data Availability

The raw data supporting the conclusions of this article will be made available by the authors, without undue reservation.
